# Integrative multivariate genomic analysis reveals shared genetic determinants and druggable targets for vascular calcification

**DOI:** 10.3389/fmed.2026.1807805

**Published:** 2026-05-15

**Authors:** Huibin Li, Gaofei Li

**Affiliations:** 1 Department of Cardiology, The First People’s Hospital of Xiaoshan District, Hangzhou, Zhejiang, China; 2 Department of General Practice, Shushan Street Community Health Service Center of Xiaoshan District, Hangzhou, Zhejiang, China

**Keywords:** drug repurposing, Mendelian randomization, multivariate genome-wide analysis, single nucleotide polymorphism, vascular calcification

## Abstract

**Background:**

Vascular calcification (VC), characterized by calcium deposition in arterial walls, is a major risk factor for cardiovascular morbidity and mortality. While genome-wide association studies (GWAS) have identified susceptibility loci for specific vascular beds, such as coronary artery calcification (CAC) and abdominal aortic calcification (AAC), single-phenotype studies may overlook pleiotropic variants. This study aims to elucidate the shared genetic architecture of CAC and AAC and translate these findings into biological insights and potential therapeutic targets.

**Methods:**

We performed a multivariate genome-wide analysis integrating summary statistics for CAC and AAC from individuals of European ancestry. To prioritize candidate genes, we applied four complementary mapping strategies, including positional mapping, multivariate set-based association test, transcriptome-wide association study, and multi-marker analysis of genomic annotation. Findings were further characterized using tissue-specific expression profiling, Gene Ontology enrichment, and cell-type specificity analysis. Therapeutic potential and safety were subsequently evaluated using OpenTargets for druggability assessment and phenome-wide association studies (PheWAS) to assess horizontal pleiotropy. Finally, experimental validation was conducted to verify the genetic findings.

**Results:**

The multivariate analysis identified seven genome-wide significant loci. Cross-referencing the four gene-mapping strategies highlighted a consensus set of robust candidate genes, with *CDKN2B* supported by all methods, and strong multi-method support for *ADAMTS7*, *PHACTR1*, and *MORF4L1*. Pathway analysis identified lipid homeostasis and cell cycle regulation as key functional modules. Cell-type specificity analysis demonstrated that candidate genes were enriched in endothelial cells. Druggability assessments identified *HDAC9* as a target for approved drugs potentially repurposed for VC, while PheWAS results suggested a predicted lack of severe genetic pleiotropy for most candidates, with the notable exception of *CDKN2A*, which showed associations with neoplasms. Quantitative real-time PCR confirmed significantly altered expression of most candidate genes, including *ADAMTS7*, *CDKN2A*, *CDKN2B*, *CXCL12*, *FHL5*, *HDAC9*, *MORF4L1*, *PDGFD*, and *PHACTR1*, in the experimental group.

**Conclusion:**

This study demonstrates that CAC and AAC share a substantial genetic basis, reinforcing the concept of VC as a systemic pathological process driven by common mechanisms. By rigorously prioritizing candidate genes and mapping them to specific cell types, we provide a comprehensive genetic map of VC and highlight potentially safe targets for future therapeutic development.

## Introduction

Vascular calcification (VC) is a highly prevalent pathological process characterized by the deposition of calcium phosphate in the vascular wall and cardiac valves, and is strongly associated with increased cardiovascular morbidity and mortality (1). Traditionally regarded as a passive and degenerative consequence of aging, VC is now recognized as a tightly regulated, cell-mediated process that shares molecular features with osteogenesis (2, 3). Despite advances in imaging-based phenotyping and experimental studies, the biological mechanisms underlying VC remain incompletely understood, and no effective pharmacological therapies are currently available to halt or reverse its progression.

Genome-wide association studies (GWAS) have provided important insights into the genetic architecture of VC by identifying susceptibility loci for specific vascular beds, most notably coronary artery calcification (CAC) (4–6) and abdominal aortic calcification (AAC) (7, 8). However, most existing studies have focused on single phenotypes in isolation. This approach may overlook genetic variants that exert pleiotropic effects across multiple vascular territories (9, 10), thereby limiting the power to detect shared biological mechanisms that broadly influence VC. Given that CAC and AAC frequently co-occur and share common risk factors (11, 12), integrating these phenotypes through multivariate genetic approaches may offer a more comprehensive understanding of the genetic determinants of VC.

In addition, translating genetic associations into biological insight remains a major challenge. Identifying candidate genes, relevant tissues and cell types, and potential therapeutic targets requires integrative analytical strategies that extend beyond conventional single nucleotide polymorphism (SNP) level analyses. Emerging tools such as multivariate GWAS (13), gene-based association tests (14, 15), transcriptome-wide association studies (TWAS) (16–18), and cell type expression-specific integration for complex traits (CELLECT) (19) provide new opportunities to systematically prioritize candidate genes and elucidate disease-relevant pathways.

In the present study, we aimed to identify shared genetic determinants underlying vascular calcification by jointly analyzing CAC and AAC using a multivariate genome-wide association framework. By integrating multiple complementary gene-mapping strategies, tissue specific expression analyses, phenome-wide association studies, and druggability assessments, we sought to prioritize robust candidate genes for VC and characterize the biological pathways and cell types through which these genes may act. Through this integrative approach, we uncover novel insights into the genetic architecture of VC and highlight candidate targets for future therapeutic development.

## Materials and methods

### Data source

**FIGURE 1:**
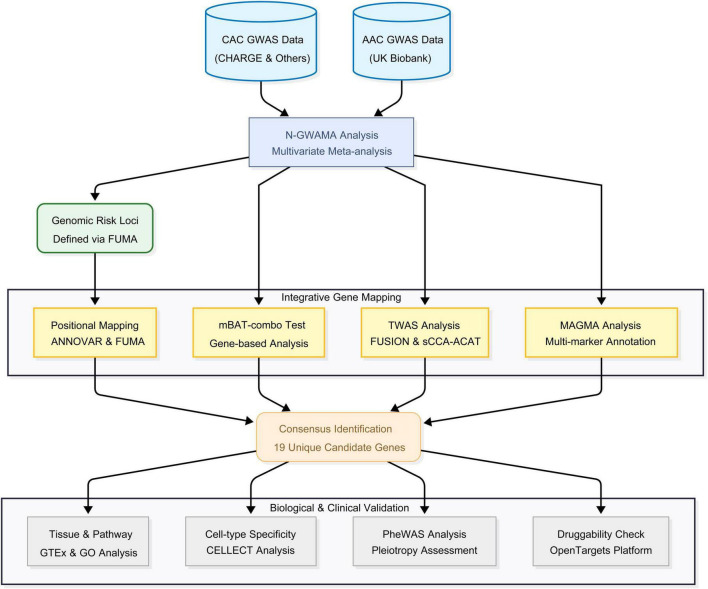
Flowchart of this study. The flowchart illustrates the comprehensive analytical framework used in this study. CAC, coronary artery calcification; AAC, abdominal aortic calcification; mBAT-combo, multivariate set-based association test-combo; TWAS, transcriptome-wide association study; sCCA-ACAT, sparse canonical correlation analysis combined with the Aggregated Cauchy Association Test; MAGMA, multi-marker analysis of genomic annotation. GO, gene ontology; CELLECT, cell type expression-specific integration for complex traits; PheWAS, phenome-wide association studies.

The overall experimental framework was shown in Figure 1. Generally, VC refers to calcium deposition in valves and vessel walls (1), but this research focused exclusively on the two primary forms of wall calcification: AAC and CAC. We acquired the latest GWAS summary statistics for these traits. CAC data originated from a CHARGE consortium meta-analysis using the Agatston scoring system (6). Although the original study involved multi-ethnic cohorts, we restricted our analysis to the 26,909 participants of European ancestry to maintain ethnic homogeneity. Conversely, AAC statistics were sourced from 31,786 European subjects in the UK Biobank imaging study. For AAC quantification, machine learning algorithms applied to lateral spine DEXA images were utilized (20) (Supplementary Table 1).

As the data are de-identified and publicly accessible, and all ethical approvals were obtained in the original studies, this secondary analysis did not require additional ethical approval from an institutional review board.

### Multivariate genome-wide analyses

We conducted a multivariate genome-wide analyses of CAC and AAC using N-GWAMA (21). N-GWAMA applies the estimates from cross-trait linkage disequilibrium score regression (LDSC) to re-weight test statistics from single-trait GWAS studies, by sample size and estimated heritability, while adjusting for genetic covariance across traits. This method enhances the detection of loci with pleiotropic associations by leveraging shared genetic architecture across traits. The newly weighted test statistics are then summated and standardized to produce a new test statistic, from which a new *p*-value can be calculated for each genetic variant. The multivariate VC-associated GWAS we get was aimed to identify an extensive set of genetic variants that broadly impact VC. By aggregating CAC and AAC into a single test statistic representing the combined latent phenotype of systemic vascular calcification, our multivariate framework (N-GWAMA) effectively performed one genome-wide scan. Thus, using the standard significance threshold is statistically appropriate without additional Bonferroni correction for multiple traits. Novel indexed variants are defined as those that were reported variants of the previous two studies.

Prior to the multivariate meta-analysis, bivariate LDSC was employed to estimate the cross-trait genetic correlation (*r_g_*) between CAC and AAC, ensuring a shared genetic basis. Additionally, the bivariate LDSC cross-trait intercept was calculated to empirically assess sample overlap and error covariance between the input cohorts. Post-analysis, univariate LDSC and the genomic inflation factor (λ_*GC*_) were computed for the multivariate summary statistics to evaluate whether potential inflation was driven by uncorrected population stratification or true polygenicity.

### Genomic risk loci definition

Genomic risk loci mapping and SNP functional annotation were performed using FUMA (v1.3.6a) (22) referencing the 1,000 Genomes Project (Phase 3) data. This platform integrates diverse external databases to generate detailed annotations. Initially, LD-based clumping was applied to isolate independent significant variants based on a *P*-value threshold of 5 × 10^–8^ and an r^2^ less than 0.6. Subsequently, we delineated independent risk loci by including all polymorphisms within a 250 kb window that demonstrated high linkage (r^2^ ≥ 0.6) with the identified independent SNPs. Finally, lead variants were characterized as the subset of independent markers exhibiting low pairwise correlation (r^2^ < 0.1). Novel indexed variants are defined as those that were >500 Kb from reported variants of the previous two studies of CAC and AAC using in our study. Variants within the 500 kb window of known loci, but representing previously unreported exact SNPs, were classified as pleiotropically novel variants.

### Gene identification

#### Positional gene mapping

Single nucleotide polymorphisms were mapped to genes using a positional mapping strategy based on ANNOVAR annotations on FUMA. The mapping criteria utilized either physical distances or functional consequences of SNPs on genes. A maximum distance of 10 kb was set to map SNPs to nearby genes. For functional-based mapping, SNPs located on the gene body (distance 0) or within upstream and downstream regions (up to 1 kb from the transcription start site or transcription end site) were included. Notably, for distances specified between >0 and <1 kb, the system defaulted to a 1 kb range due to annotation resolution. For SNPs located in regions where multiple genes overlapped, the system prioritized the gene mapping based on the most deleterious functional consequence.

#### Multivariate set-based association test

We performed a gene-based analysis using the multivariate set-based association test (mBAT)-combo implemented in the GCTA software (version 1.94.1) (23). The analysis was based on GWAS summary statistics and incorporated LD information from the European subsample (*n* = 503) of the 1,000 Genomes Project Phase 3, with an LD cutoff set at 0.9 as default in fastBAT. Gene mapping was conducted using genome build hg19, covering 19,899 protein-coding genes. By integrating GWAS summary data with LD reference data, mBAT-combo provides a more comprehensive assessment of genetic influences on complex traits, especially when multiple SNPs within a gene or region collectively affect a phenotype. Significance was defined by a Benjamini-Hochberg adjusted false discovery rate (FDR) below 0.05.

#### Transcriptome-wide association study

Utilizing the FUSION framework, we conducted a transcriptome-wide association study (TWAS) (17) to detect genes exhibiting cis-regulated links to VC. The procedure began by building prediction algorithms for gene expression using expression quantitative trait loci (eQTL) datasets. These models were then applied to estimate expression profiles across extensive genotyped cohorts. We subsequently tested for statistical correlations between the imputed expression values and the disease phenotype. Our approach incorporated pre-calculated weights from arterial tissues (specifically GTEx V8 coronary, tibial, and aorta arteries). Furthermore, a cross-tissue expression signature for VC was established using sparse canonical correlation analysis combined with the Aggregated Cauchy Association Test (sCCA-ACAT) (24). Significance was defined by a Benjamini-Hochberg adjusted FDR below 0.05.

#### Multi-marker analysis of genomic annotation analysis

To isolate specific genes linked to VC, we utilized the multi-marker analysis of genomic annotation (MAGMA) tool (v1.08) (25). Initially, variants were assigned to protein-coding loci referencing hg19 coordinates. We extended the boundaries by 10 kb on both ends to capture potential regulatory elements. LD patterns were modeled using European samples from the 1,000 Genomes Phase 3 dataset. Significance values were generated via the SNP-wise mean method, which combines the collective effects of all variants within a gene. Finally, significance was defined by a Benjamini-Hochberg adjusted FDR below 0.05.

### Tissue type identification and enrichment analyses

For the complete set of unique candidate genes prioritized across all four mapping strategies, we utilized the GENE2FUNC module within FUMA to assess tissue specificity, referencing expression profiles across 30 general tissues from the GTEx v8 database (26). Furthermore, this tool was employed to perform functional enrichment tests using the same set of candidate genes. Our assessment covered Gene Ontology (GO) categories, specifically biological processes, molecular functions, and cellular components, prioritizing pathways that exhibited an adjusted *P*-value lower than 0.05.

### Cell-type specificity analysis

To prioritize cell types potentially involved in the etiology of VC, we applied the cell type expression-specific integration for complex traits (CELLECT) framework (27) to integrate GWAS summary statistics with cell-type-specific expression information. Cell-type expression-specific likelihood estimator for gene expression specificity (CELLEX), a computational toolkit used alongside CELLECT, was employed to quantify how specifically each gene is expressed in individual cell types based on single-cell RNA sequencing (scRNA-seq) data. Briefly, CELLEX first generated a continuous expression-specificity score (ESμ) for each cell type by combining four complementary metrics, after which MAGMA was used to calculate gene-level association statistics by aggregating SNP signals within genes and their 100-kb flanking regions while accounting for linkage disequilibrium. After adjustment for default MAGMA covariates, linear regression was performed to test whether genes specifically expressed in a given cell type showed stronger genetic associations with VC. We used the Tabula Muris resource, which contains transcriptomic profiles from Mus musculus spanning a wide range of organs and tissues (28), and statistical significance was defined using an FDR-corrected threshold of *P* < 0.05.

### Phenome-wide association analysis

To evaluate potential side effects and horizontal pleiotropy of candidate targets, we utilized the AstraZeneca phenome-wide association study (PheWAS) Portal (29). This analysis involved running a PheWAS across 17,361 binary disease traits, leveraging exome sequencing records from 269,171 European subjects in the UK Biobank. We applied the portal’s standard significance cutoff of 1 × 10^–8^ to minimize FDR.

### Druggability

To assess the therapeutic viability of the identified genes, we utilized the OpenTargets platform (30). Although this database originally sorts targets into nine “buckets” based on development phase and modality, we consolidated them into four simplified categories (31). The first represents approved treatments (Bucket 1). The second encompasses agents in clinical trials (Buckets 2 and 3). The third aggregates preclinical and theoretical targets (combining Buckets 4 and 5 for antibodies/small molecules and Buckets 6–8 for small molecules). Finally, the fourth group contains all remaining undruggable targets.

### Validation experiment

To establish an *in vitro* calcification model, human aortic vascular smooth muscle cells (VSMCs; CRL-1999) obtained from ATCC (Manassas, VA, United States) were exposed to a phosphate-enriched environment containing 3 mM inorganic phosphate (Pi) for 10 days, with media replacement performed every 48 h. Prior to induction, cells at passages 3–8 were maintained under standard conditions in Dulbecco’s modified Eagle’s medium (DMEM; Thermo Fisher Scientific, United States) supplemented with 10% fetal bovine serum (FBS; Gibco, United States). For routine culture, a reduced-serum maintenance medium was used, consisting of DMEM with 2% FBS and antibiotics (100 U/mL penicillin and 100 μg/mL streptomycin; Thermo Fisher Scientific, United States).

Human VSMCs was induced by calcifying medium (CM) and growth medium (GM). Following treatment, total RNA was isolated using TRIzol reagent and reverse-transcribed into cDNA with the PrimeScript RT system (Takara, Japan). Quantitative real-time polymerase chain reaction (qPCR) was then performed to evaluate the expression levels of selected candidate genes. Reactions were carried out using a SYBR Green-based detection system (Vazyme) under conditions recommended by the manufacturer. All primers were synthesized by Sangon Biotech (Shanghai, China). Gene expression levels were normalized to β-actin and calculated using the comparative Ct (2^–ΔΔCt^) method. A Student’s *t*-test was applied to analyze the PCR results, and a *P*-value < 0.05 was considered statistically significant.

## Results

### Multivariate genome-wide analyses and quality control

To investigate the shared genetic architecture underlying systemic vascular calcification, we conducted a multivariate genome-wide analyses integrating two primary clinical manifestations from 60,441 individuals of European ancestry (Figure 1 and Supplementary Table 1). Before performing the genome-wide analyses, we estimated the genetic correlation between the two traits using LDSC. The trait pair exhibited a strong and highly significant genetic correlation (*r_g_* = 0.8125, SE = 0.1547, *P* = 1.50 × 10^–7^), empirically justifying their integration into a multivariate framework. Furthermore, regarding sample overlap, the summary statistics for CAC and AAC were derived from completely independent cohorts. We confirmed the absence of sample overlap using bivariate LDSC, which yielded a cross-trait intercept of approximately zero (Intercept = 0.001, SE = 0.0051). Consequently, the environmental and error covariance between the two datasets was negligible.

**FIGURE 2:**
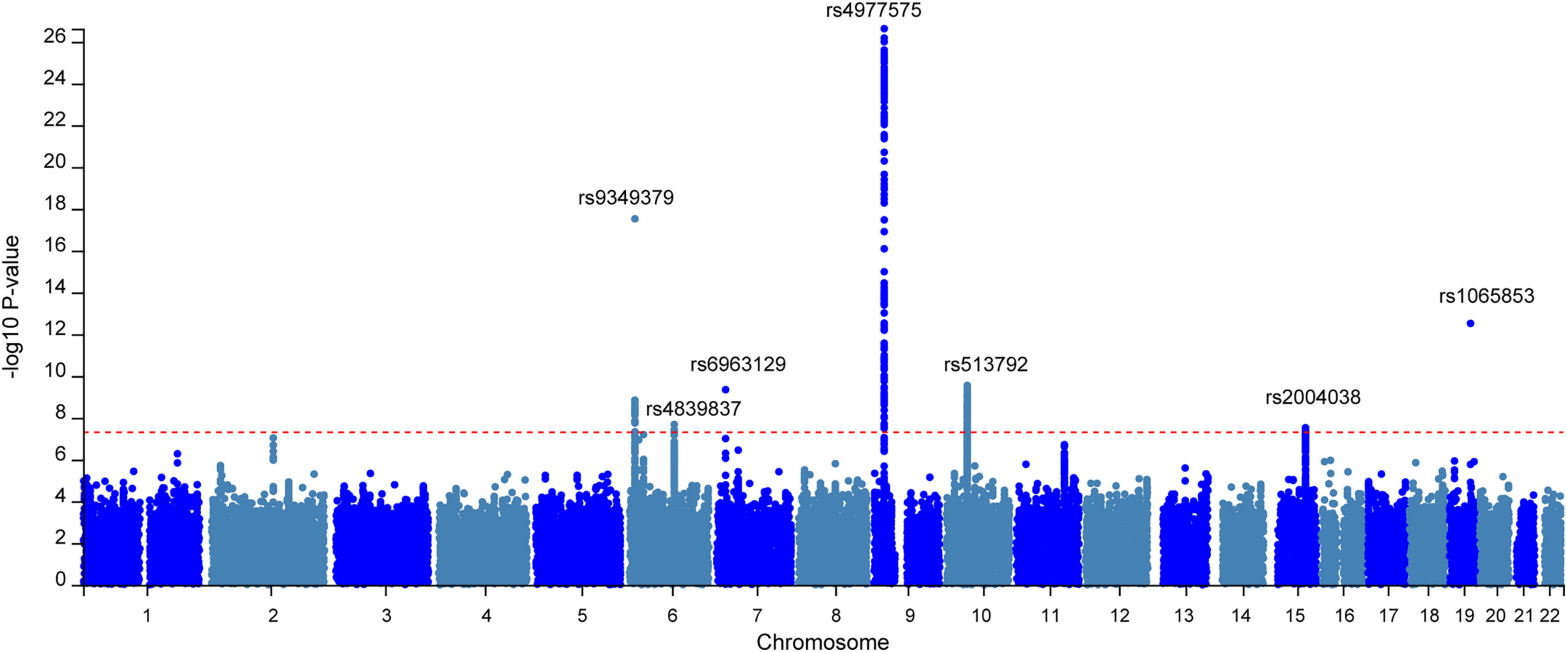
Manhattan plots of the genome-wide association studies (GWAS). Manhattan plot showing the –log_10_ *p*-value of association for each SNP from the GWAS plotted on the vertical axis against genomic position on the horizontal axis. The red dotted line corresponds to the genome-wide significance threshold (*P* < 5 × 10^– 8^) and marked SNPs was the relevant lead SNPs of risk locus.

Following these rigorous quality controls, our N-GWAMA successfully identified seven distinct genomic loci reaching genome-wide significance (*P* < 5 × 10^–8^) (Figure 2). Within these regions, seven independent lead variants were isolated using LD clumping (r^2^ < 0.1 based on the 1000G Phase 3 panel) (Table 1 and Supplementary Table 2). Specifically, the seven genome-wide significant loci were classified into three categories. First, one novel locus on chromosome 6 (rs4839837) was identified and undetected in single-trait analyses. Second, three pleiotropically novel variants (rs6963129, rs513792, rs2004038) were identified, which lie within 500 kb of known regions. Finally, three known loci were replicated. The genomic inflation factor (λ_*GC*_) for the multivariate GWAS was 1.06, and the univariate LDSC intercept was 1.03 (SE = 0.01). This indicates that the observed statistical signal is predominantly driven by true polygenicity rather than uncorrected population stratification (Supplementary Figures 1, 2).

**TABLE 1:** Genomic risk loci of GWAS.

Genomic locus	Index SNP	Chromosome	Position	*P*-value	Signal type
1	rs9349379	6	12903957	3.03 × 10^–18^	Known variants
2	rs4839837	6	96908706	2.11 × 10^–8^	Novel variants
3	rs6963129	7	19046177	4.58 × 10^–10^	Pleiotropically novel variants
4	rs4977575	9	22124744	2.38 × 10^–27^	Known variants
5	rs513792	10	44744252	2.86 × 10^–10^	Known variants
6	rs2004038	15	79071406	3.03 × 10^–8^	Pleiotropically novel variants
7	rs1065853	19	45413233	3.07 × 10^–13^	Pleiotropically novel variants

SNP, single nucleotide polymorphism; GWAS, genome-wide association studies.

### Gene identification

**FIGURE 3:**
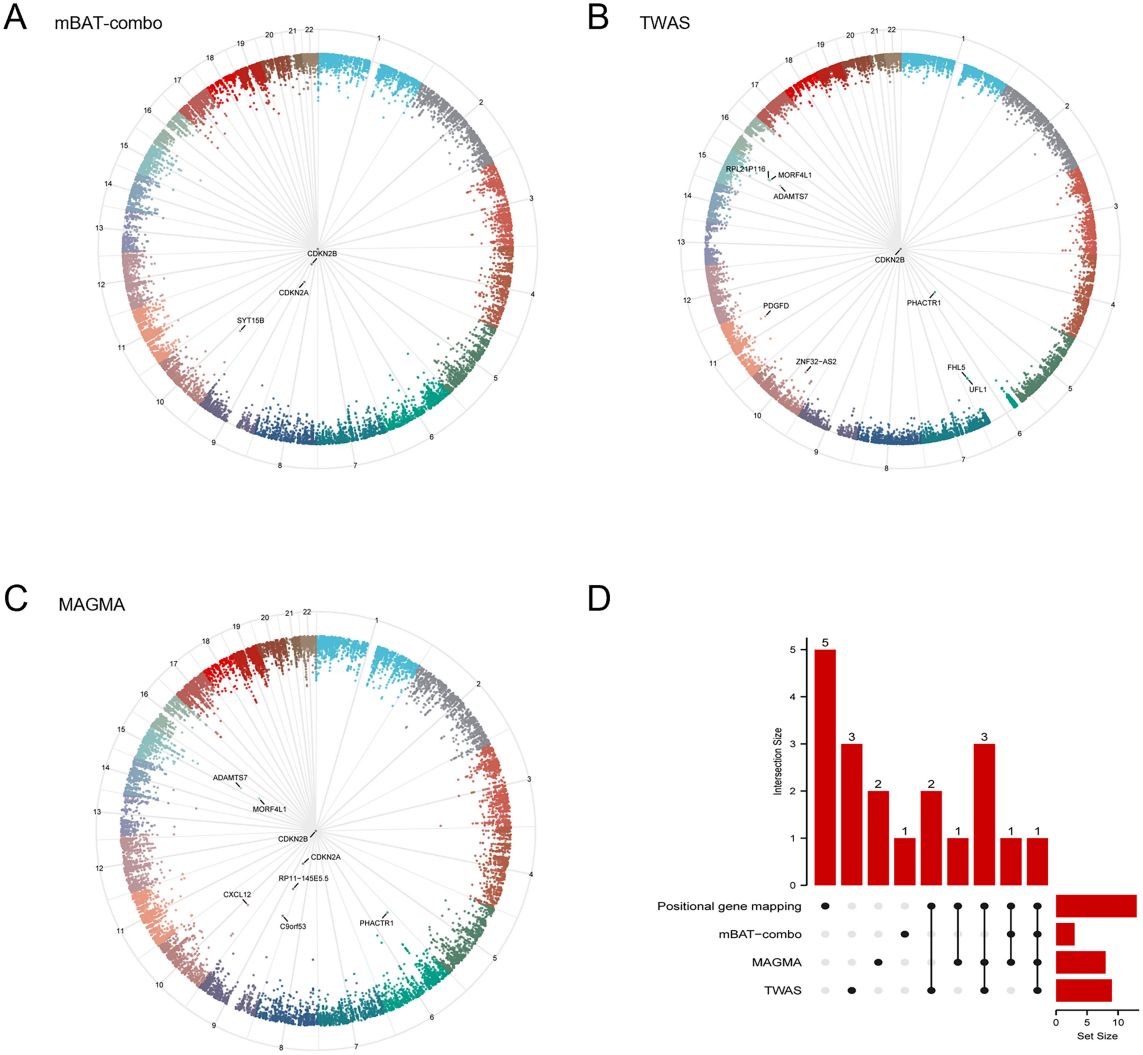
Integrative gene mapping and consensus gene identification. **(A–C)** Circular plots visualizing the genomic distribution of significant loci and their associated genes identified through different mapping approaches. The outer rings represent chromosomes, and points indicate significant association signals. **(D)** UpSet plot illustrating the intersection of candidate genes identified by the four distinct mapping strategies. mBAT-combo, multivariate set-based association test-combo; TWAS, transcriptome-wide association study; MAGMA, multi-marker analysis of genomic annotation.

To prioritize candidate genes, we implemented four complementary mapping strategies. First, positional mapping based on deleterious SNPs identified 14 genes across seven genomic risk loci (Table 2). Independently, the mBAT-combo test prioritized three genes (*CDKN2A, CDKN2B, SYT15B*) (Figure 3A and Supplementary Table 3). In parallel, TWAS analysis implicated nine genes (*ADAMTS7, CDKN2B, FHL5, MORF4L1, PDGFD, PHACTR1, RPL21P116, UFL1, ZNF32-AS2*) (Figure 3B and Supplementary Table 4) that surpassed the combined significance threshold. Finally, MAGMA methods highlighted eight genes (*ADAMTS7, C9orf53, CDKN2A, CDKN2B, CXCL12, MORF4L1, PHACTR1, RP11-145E5.5*) (Figure 3C and Supplementary Table 5) meeting the same stringent criteria.

**TABLE 2:** The results of positional mapping.

Gene symbol	Chromosome	Start	End	Type	Entrez ID	HUGO	Genomic locus
*PHACTR1*	6	12717893	13288645	Protein coding	221692	*PHACTR1*	1
*UFL1*	6	96969471	97003152	Protein coding	23376	*UFL1*	2
*FHL5*	6	97010424	97064512	Protein coding	9457	*FHL5*	2
*HDAC9*	7	18126572	19042039	Protein coding	9734	*HDAC9*	3
*RP11-145E5.5*	9	21802635	22032985	Protein coding	NA	NA	4
*C9orf53*	9	21967137	21967738	Protein coding	NA	*C9orf53*	4
*CDKN2A*	9	21967751	21995300	Protein coding	1029	*CDKN2A*	4
*CDKN2B*	9	22002902	22009362	Protein coding	1030	*CDKN2B*	4
*CHRNB4*	15	78916461	79020096	Protein coding	1143	*CHRNB4*	6
*ADAMTS7*	15	79051545	79103773	Protein coding	11173	*ADAMTS7*	6
*MORF4L1*	15	79102829	79190475	Protein coding	10933	*MORF4L1*	6
*TOMM40*	19	45393826	45406946	Protein coding	10452	*TOMM40*	7
*APOE*	19	45409011	45412650	Protein coding	348	*APOE*	7
*APOC1*	19	45417504	45422606	Protein coding	341	*APOC1*	7

The four prioritization methods combined prioritized 19 unique genes (Supplementary Table 6). To prioritize the most plausible candidate genes, we examined the intersection of these mapping sets. The analysis highlighted a consensus set of genes supported by multiple lines of evidence (Figure 3D). *CDKN2B* exhibited the highest level of support, lying at the intersection of mBAT-combo, TWAS, MAGMA and positional gene mapping results. Additionally, multi-method overlaps were observed for *ADAMTS7, MORF4L1*, and *PHACTR1* (shared by TWAS, MAGMA, and positional gene mapping) and *CDKN2A* (shared by mBAT-combo and MAGMA and positional gene mapping), suggesting shared genetic architectures captured by these distinct methods.

### Tissue specificity gene expression and pathway enrichment analyses

**FIGURE 4:**
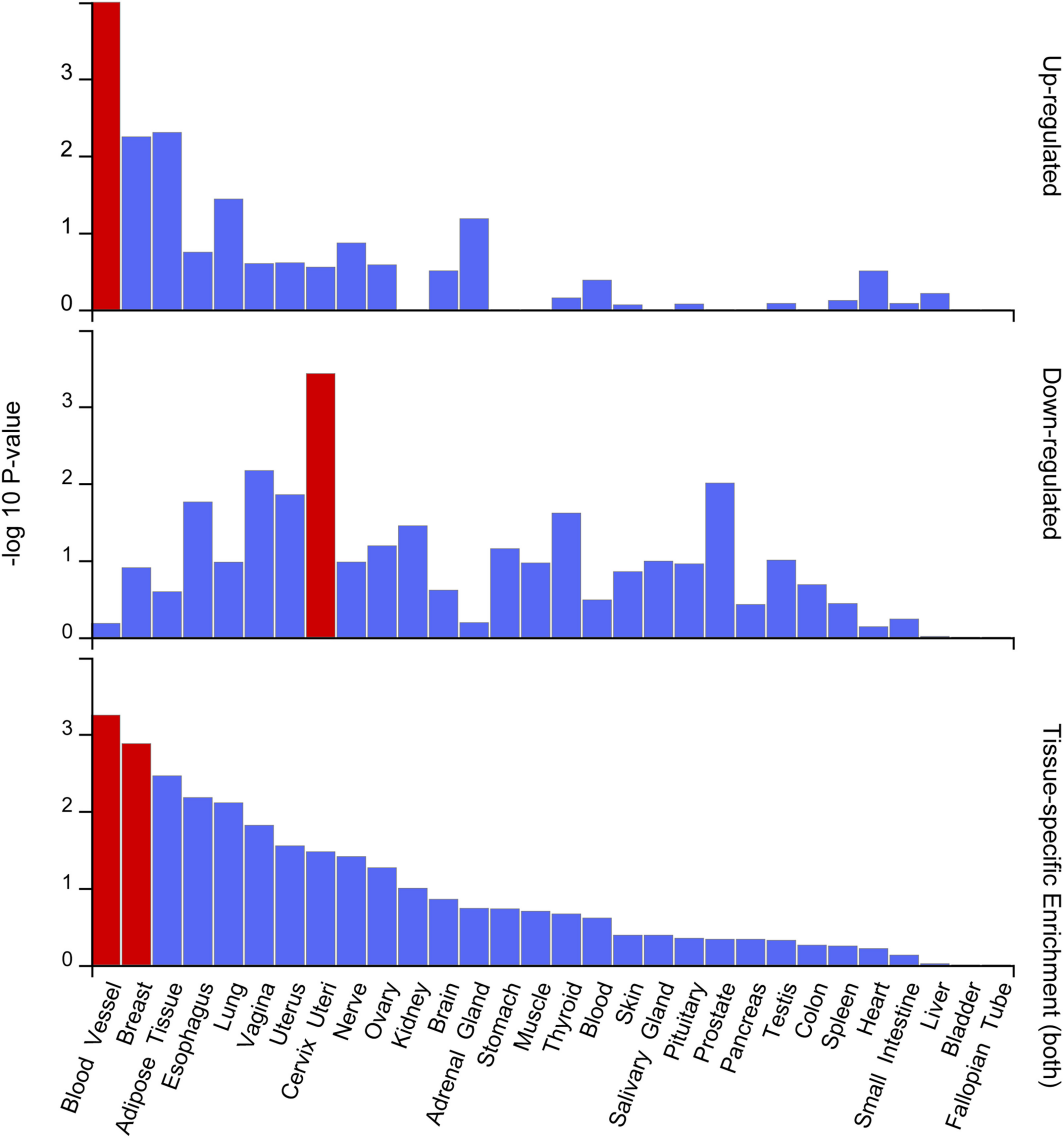
Tissue enrichment analysis of candidate genes. Tissue enrichment analysis of candidate genes in 30 GTEx V8 tissues. The red bars represent tissues in which the gene set is differentially expressed. The blue bars represent tissues in which the gene set is not differentially expressed.

**FIGURE 5:**
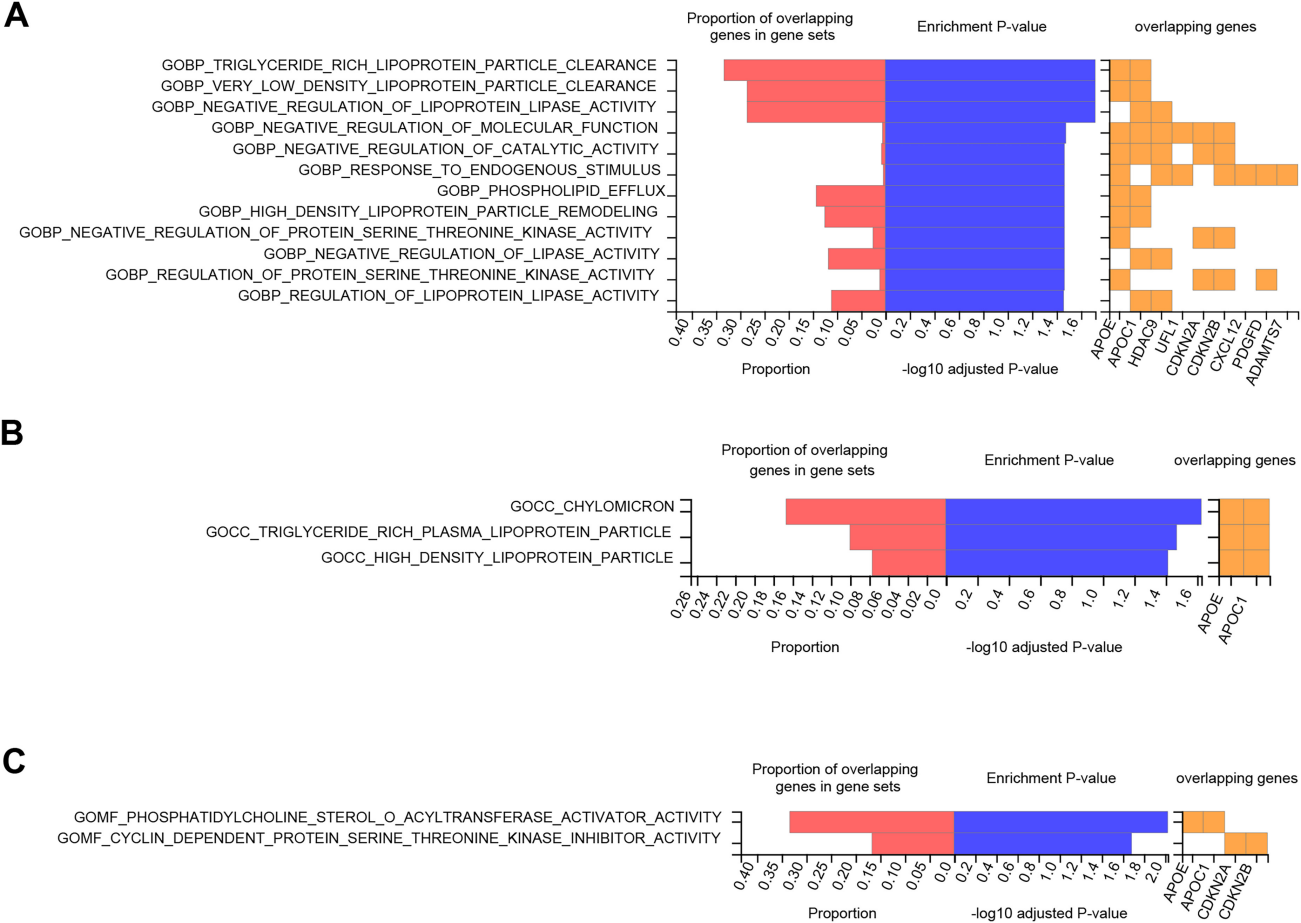
Functional enrichment analysis of identified genes. The graphs summarize the significant GO terms enriched among the 19 identified genes. **(A)** GOBP **(B)** GOCC **(C)** GOMF. For each panel, the left bars display the proportion of overlapping genes (red) and the enrichment significance (–log_10_ adjusted *P*-value, blue). The heatmap on the right indicates the specific genes driving the enrichment in each pathway. GO, gene ontology; GOBP, GO biological processes; GOCC, GO cellular components; GOMF, GO molecular functions.

To assess the tissue specificity of the 19 prioritized candidate genes, we evaluated their relative abundance across various normal human tissues and regions. The analysis revealed that this gene set was significantly enriched predominantly in blood vessel tissues (Figure 4). GO enrichment analysis highlighted two distinct functional modules driven by specific risk loci (Figures 5A–C). The first module showed significant enrichment in lipid homeostasis pathways, particularly triglyceride-rich lipoprotein particle clearance and chylomicron components. The second module was characterized by cell cycle regulatory terms, specifically negative regulation of protein serine/threonine kinase activity and cyclin-dependent protein serine/threonine kinase inhibitor activity.

### Cell-type specificity analysis

**FIGURE 6:**
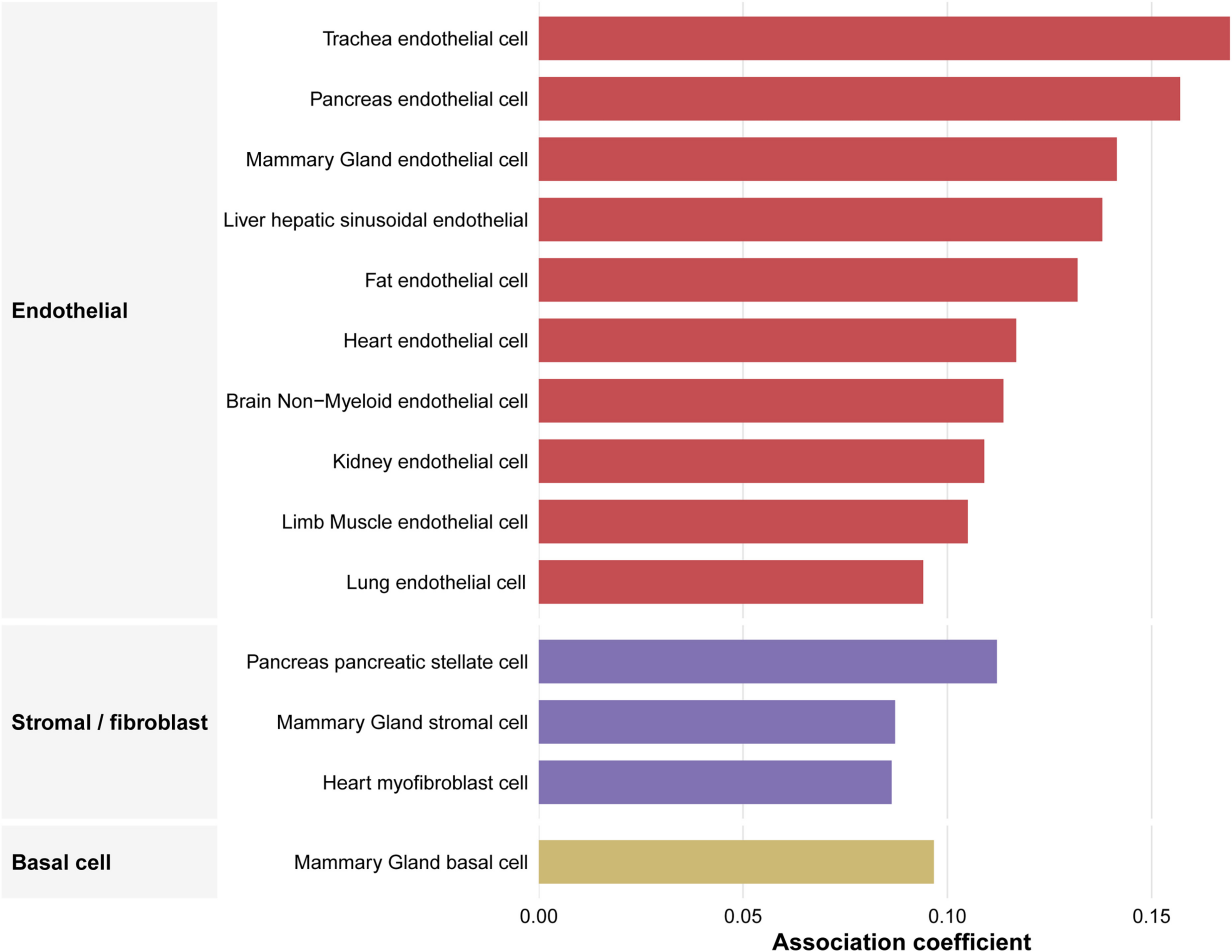
CELLECT-based cell-type specificity analysis for vascular calcification. Faceted bar plot of significant positively associated cell populations identified from VC GWAS data. Cell populations are grouped by major cell-type categories and ranked by association coefficient. Endothelial cells constituted the predominant category among positively associated cell populations. VC, vascular calcification.

Using VC GWAS summary statistics in the CELLECT analysis, we observed that the association pattern was predominantly driven by endothelial cells. At the significance threshold, 14 cell populations showed positive associations with VC, among which 10 belonged to the endothelial lineage (Figure 6 and Supplementary Table 7). The strongest signal was identified in endothelial cells, with tracheal endothelial cells showing the most significant association (*P* = 5.26 × 10^–4^), followed by liver sinusoidal endothelial cells, pancreatic endothelial cells, fat endothelial cells, and brain non-myeloid endothelial cells. In addition to endothelial populations, a smaller number of stromal-related cell types, including basal cells, stellate cells, stromal cells, and myofibroblasts, also showed nominal associations (Figure 6 and Supplementary Table 7). Overall, these results indicate that VC-associated genetic signals are preferentially enriched in endothelial cells, with comparatively weaker contributions from stromal-related populations.

### PheWAS results

We conducted a gene-level PheWAS via the AstraZeneca PheWAS Portal to evaluate the pleiotropic effects of the 14 available candidate genes. With the exception of *CDKN2A*, none of the genes displayed significant trait associations (*P* < 1 × 10^–^8), indicating limited horizontal pleiotropy and suggesting a potentially favorable genetic safety profile (Supplementary Table 8). In contrast, *CDKN2A* was significantly associated with neoplasms, which highlights potential on-target effects associated with its modulation.

### Druggability results

Nine genes were predicted to be a drug target for treatment (Supplementary Table 9). *CHRNB4* and *HDAC9* were grouped as approved drugs (Supplementary Table 9). There are known drugs available for repurposing that target all the genes mentioned above. *CXCL12* was grouped as clinical development-stage drugs (Supplementary Table 9). *APOC1*, *APOE, MORF4L1*, *PDGFD*, *PHACTR1*, *TOMM40* were grouped as preclinical and predicted therapeutic drugs. Drug information for known drugs of approved drugs and clinical development-stage drugs is listed in Supplementary Table 10.

### Validation experiment

**FIGURE 7:**
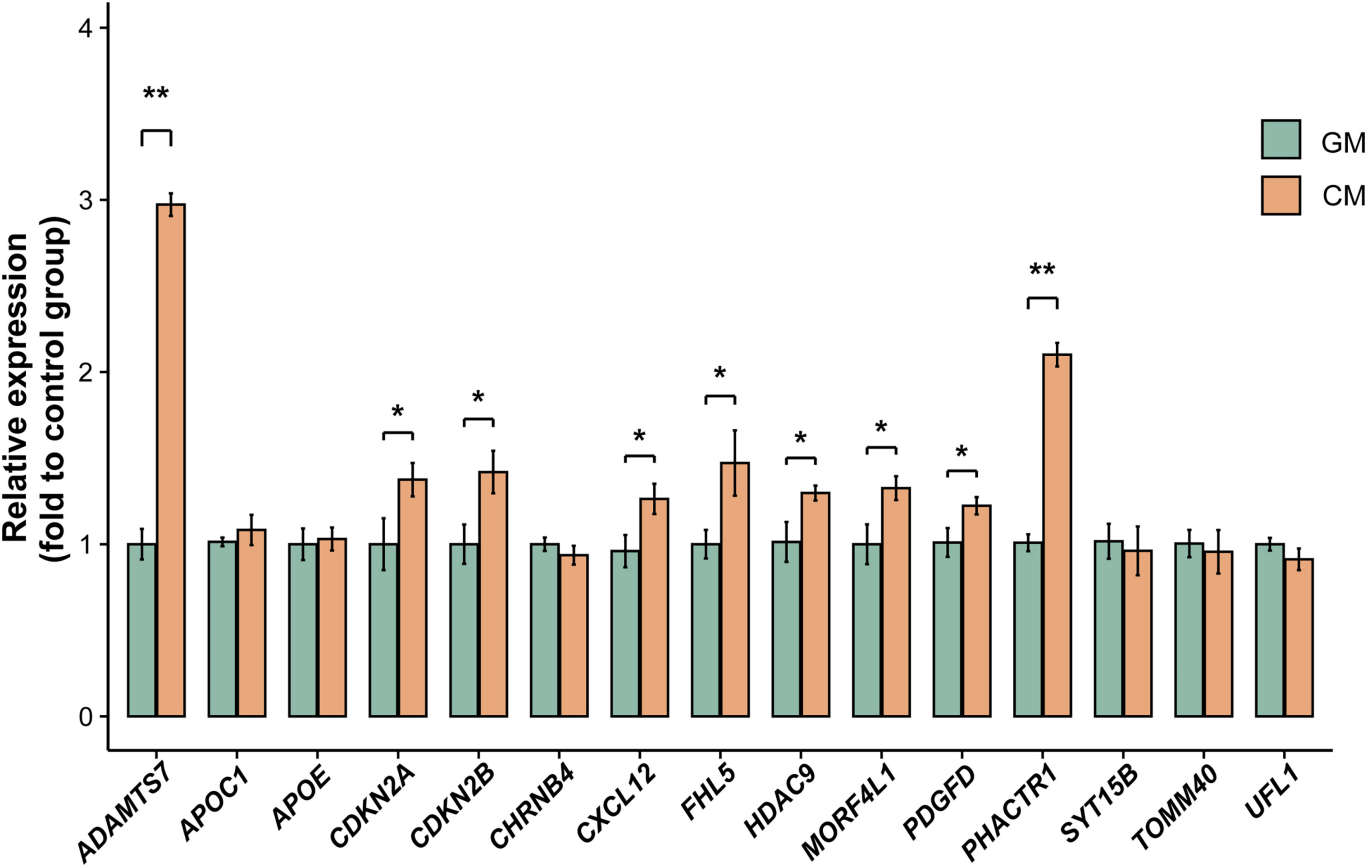
mRNA levels of relevant genes between the GM and CM groups in human vascular smooth muscle cells (VSMCs). Significant expression changes in 9 genes in the CM group compared to the GM group. Values are expressed as mean ± SD of three individual experiments. *P < 0.05, **P < 0.01. *n* = 3 for each group. GM, growth medium; CM, calcifying medium; SD, standard deviation.

To further verify the robustness of our findings, we compared the mRNA expression levels of relevant genes between the GM and CM groups in human VSMCs. Primers were available for 15 candidate genes, and their sequences are listed in Supplementary Table 11. qPCR analysis was subsequently conducted to assess the expression of these 15 VC-related genes. Among them, nine genes, including *ADAMTS7*, *CDKN2A*, *CDKN2B*, *CXCL12*, *FHL5*, *HDAC9*, *MORF4L1*, *PDGFD*, and *PHACTR1*, showed significant expression changes in the CM group relative to the control group (Figure 7).

## Discussion

In this study, we employed a multivariate genome-wide association framework to investigate the shared genetic architecture of CAC and AAC. By integrating multidimensional post-GWAS analyses, including consensus gene mapping (mBAT-combo, TWAS, MAGMA, positional), tissue-specific expression profiling, cell-type specificity analysis and PheWAS, we systematically prioritized robust candidate genes and computationally predicted druggable targets for VC. Our findings underscore the systemic nature of VC, suggest a potential contribution of endothelial-cell-related programs to VC susceptibility, and identify potential therapeutic avenues with fewer predicted pleiotropic side effects.

Traditionally, VC has been studied in specific vascular beds in isolation (32, 33). However, our multivariate meta-analysis demonstrates that CAC and AAC share a substantial genetic basis, supporting the hypothesis that VC is a systemic pathological process driven by common biological mechanisms rather than merely a local degenerative phenomenon. By utilizing N-GWAMA, we effectively leveraged the genetic covariance between traits to enhance statistical power, allowing us to capture pleiotropic loci that might be missed in single-trait analyses. This aligns with previous findings suggesting that despite heterogeneity in embryonic originand hemodynamic stress between coronary and abdominal vessels, the fundamental osteogenic signaling pathways are conserved (34–36). The identification of seven multi-trait risk loci reinforces the utility of multivariate approaches in dissecting the complex genetics of cardiovascular pathologies.

A major strength of our study is the use of four complementary gene-mapping strategies to overcome the limitations of distance-based mapping. The convergence of evidence from positional mapping, mBAT-combo, TWAS, and MAGMA highlighted a consensus set of genes, with *CDKN2B* receiving the strongest support (identified by all four methods). *CDKN2B*, located at the 9p21 locus, is a well-established regulator of the cell cycle (37, 38). Its robust association in our multivariate model supports a potential role in VC, potentially through the regulation of vascular wall cell proliferation and senescence, which are precursor steps to osteochondrogenic transdifferentiation (39, 40). Specifically, variants at this locus have been previously associated with increased calcified plaques in human arterial tissues (41), and recent *in vitro* models have provided compelling evidence for a causal linkage between this locus and the osteochondrogenic phenotypic shift (42). While these pivotal studies established the mechanistic importance of *CDKN2B* in localized vascular calcification, our present analysis significantly extends this knowledge. By identifying *CDKN2B* as the top consensus gene in a multivariate framework, we demonstrate its robust, pleiotropic effect across distinct vascular beds (coronary and abdominal aorta) at the genetic level. Furthermore, its consistent emergence across four independent computational mapping strategies serves as an encouraging positive control, lending confidence to our analytical pipeline and aligning with the hypothesis that systemic VC may be closely linked to aberrations in cell cycle regulation.

Furthermore, our identification of *ADAMTS7* and *PHACTR1* across multiple methods highlights their importance. The consistency across distinct analytical frameworks significantly increases the confidence that these may represent functional drivers rather than mere passengers in LD. *PHACTR1* has been implicated in cytoskeletal organization, cell migration, inflammatory regulation, and vascular tone modulation (43), and has also been identified as a susceptibility locus for coronary artery disease and related vascular phenotypes (44). These features suggest that *PHACTR1* may contribute to VC through effects on vascular cell phenotype and local inflammatory responses (45, 46). *ADAMTS7*, an extracellular matrix-remodeling protease (47), has been consistently implicated in atherosclerosis and vascular injury (48), and may promote calcification by altering matrix homeostasis and creating a microenvironment permissive for mineral deposition (49). Besides, *HDAC9* appear to be particularly relevant to VC pathobiology. *HDAC9*, a member of the class IIa histone deacetylase family (50), is a key epigenetic regulator of gene transcription and has been linked to pro-calcific and pro-inflammatory signaling in vascular disease (51). Prior studies indicate that *HDAC9* may facilitate osteogenic remodeling by repressing anti-calcific pathways and enhancing inflammatory activity, supporting its candidacy as a potential therapeutic target (52, 53). Taken together, these genes converge on several core processes relevant to VC, including vascular remodeling, inflammatory activation, extracellular matrix reorganization, and osteogenic transition, reinforcing their biological plausibility as candidate drivers of disease.

Translating genetic signals to cell-specific mechanisms is critical. Our CELLECT analysis of VC GWAS data indicated that the suggestive enrichment pattern was predominantly driven by endothelial cells, whereas only a smaller number of stromal-related populations showed weaker nominal associations. This pattern is biologically plausible, as vascular calcification is now widely recognized as an active, tightly regulated process rather than a passive deposition of calcium salts, involving coordinated phenotypic alterations across multiple vascular wall cell types (54, 55). In particular, accumulating evidence suggests that endothelial cells are not merely passive bystanders during calcification but may actively contribute to disease initiation and progression through endothelial dysfunction, osteogenic reprogramming, and endothelial-to-mesenchymal transition (EndMT) (56, 57). Experimental studies have further shown that endothelial-lineage cells can serve as a source of osteoprogenitor-like cells in calcified vascular lesions, and that EndMT is enhanced in calcifying settings such as diabetes and inflammation (56, 58, 59). The additional nominal signals observed in stromal and myofibroblast-related populations are also noteworthy, because fibroblast-lineage cells exhibit substantial phenotypic plasticity and have been implicated in extracellular matrix remodeling, fibrosis, and calcific lesion formation (60). Collectively, these findings support a model in which genetic susceptibility to VC may preferentially converge on endothelial injury and endothelial phenotypic switching.

Our pathway enrichment analysis identified two primary biological modules: lipid homeostasis and cell cycle regulation. The enrichment of lipid-related pathways (*APOE*, *APOC1*) is consistent with the clinical observation that dyslipidemia is a major risk factor for VC (61, 62). Lipids, particularly oxidized LDL, can trigger osteogenic signaling in the vascular wall (63). Concurrently, the enrichment of cell cycle pathways (driven by *CDKN2A/B*) points to the proliferative aspect of plaque formation (64). The dual involvement of these pathways suggests that VC therapeutics may need to simultaneously target metabolic dysregulation and aberrant cell proliferation to be effective.

Finally, a critical objective of this study was to bridge the gap between discovery and translation. Our druggability assessment identified *HDAC9* and *CHRNB4* as targets of approved drugs, and *CXCL12* as a target for drugs in clinical development. *HDAC9* is a histone deacetylase involved in epigenetic regulation, it may influence pro-calcific signaling and therefore represents a plausible candidate for therapeutic targeting (65). HDAC inhibitors, already used in oncology, could potentially be repurposed for VC, although their systemic effects require careful evaluation (66). To computationally estimate potential safety concerns, our PheWAS analysis evaluated horizontal pleiotropy. Most candidate genes showed limited pleiotropic associations, suggesting that targeting them might have a low risk of off-target side effects. However, *CDKN2A* showed a significant association with neoplasms (67–69). This is a crucial finding, as *CDKN2A* encodes p16INK4a, a tumor suppressor (70). While enhancing its expression might protect against VC by limiting cell proliferation, it could theoretically alter cancer risk, or conversely, inhibiting it could promote tumorigenesis (71, 72). Conversely, genes like *ADAMTS7* or *PHACTR1* did not show such adverse associations, making them potentially safer therapeutic targets from a genetic pleiotropy perspective, though rigorous preclinical safety evaluations are still necessary.

## Limitations

First, the GWAS data were predominantly from individuals of European descent, limiting the generalizability of our findings to other ancestries. Second, the cell-type prioritization analysis was based on the Tabula Muris mouse atlas rather than vascular tissue-specific human single-cell data. Therefore, the observed endothelial enrichment should be interpreted cautiously, as species differences and the broad composition of the reference atlas may limit direct translation to human VC. Future studies using dedicated human vascular calcification single-cell datasets will be necessary to validate and refine these findings. Finally, it is important to acknowledge that our findings regarding gene prioritization, PheWAS-based safety profiles, and druggability are based on *in silico* predictive models and are inherently hypothesis-generating while we successfully provided initial *in vitro* validation confirming the significant transcriptional dysregulation of candidate genes during VSMC calcification. Future rigorous *in vivo* animal models and mechanistic knockout studies are required to establish definitive causality and to evaluate the actual therapeutic efficacy and clinical safety of targeting these loci before clinical translation.

## Conclusion

In summary, our multivariate genomic analysis provides a comprehensive map of the genetic determinants of vascular calcification. By systematically prioritizing candidate genes like *CDKN2B, PHACTR1*, and *ADAMTS7*, and computationally mapping them to specific vascular cell types and biological pathways, we provide compelling genetic evidence supporting the role of lipid homeostasis and cell cycle regulatory in VC. While the identification of computationally predicted druggable targets with limited genetic pleiotropy offers a theoretical roadmap for precision therapeutics, these associations remain predictive. Rigorous *in vitro* and *in vivo* experimental validations are essential to confirm their mechanistic roles and therapeutic viability before clinical translation.

## Data Availability

The original contributions presented in this study are included in this article/Supplementary material, further inquiries can be directed to the corresponding author.
