# Supplementary material for: Integrative multivariate genomic analysis reveals shared genetic determinants and druggable targets for vascular calcification
Source: Front Med (Lausanne). 2026 May 15;13:1807805. doi: 10.3389/fmed.2026.1807805 (PMC13218890; doi:10.3389/fmed.2026.1807805)

**Supplementary Figure 1. Quantile-Quantile plot of the genome-wide association study of vascular calcification.**

In the Quantile-Quantile plot, the x-axis represents the expected -log_10_ P-value, and the y-axis represents the observed -log10 P-value. The scatter points represent the distribution of P-values for all SNPs. The red dashed line indicates the theoretical null distribution (observed = expected). If the scatter points deviate from the dashed line, it suggests potential genetic association signals.


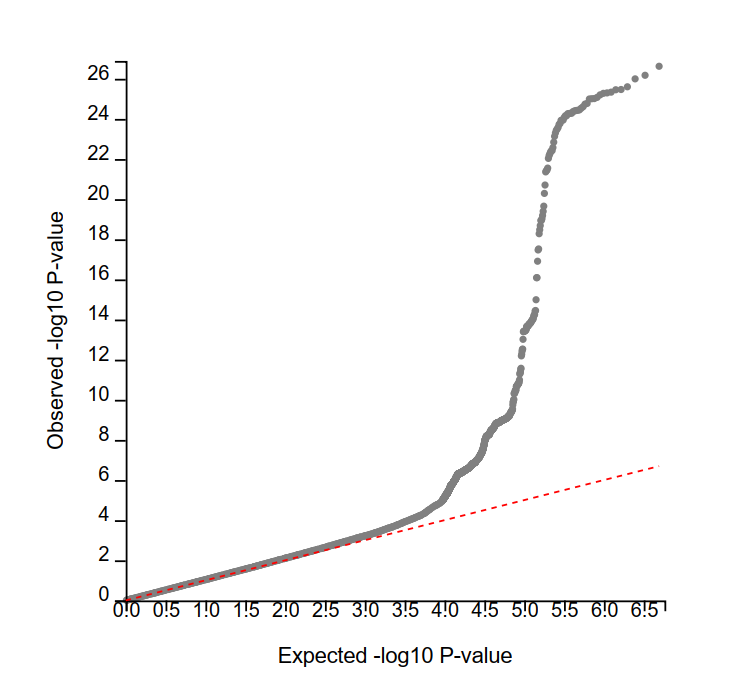


**Supplementary Figure 2. Manhattan plots of the GWAS**

Manhattan plots (A for AAC and B for CAC) showing the -log_10_ p-value of association for each SNP from the GWAS plotted on the vertical axis against genomic position on the horizontal axis. The red dotted line corresponds to the genome-wide significance threshold (P < 5×10^-8^).

Abbreviation: CAC, coronary artery calcification; AAC, abdominal aortic calcification.


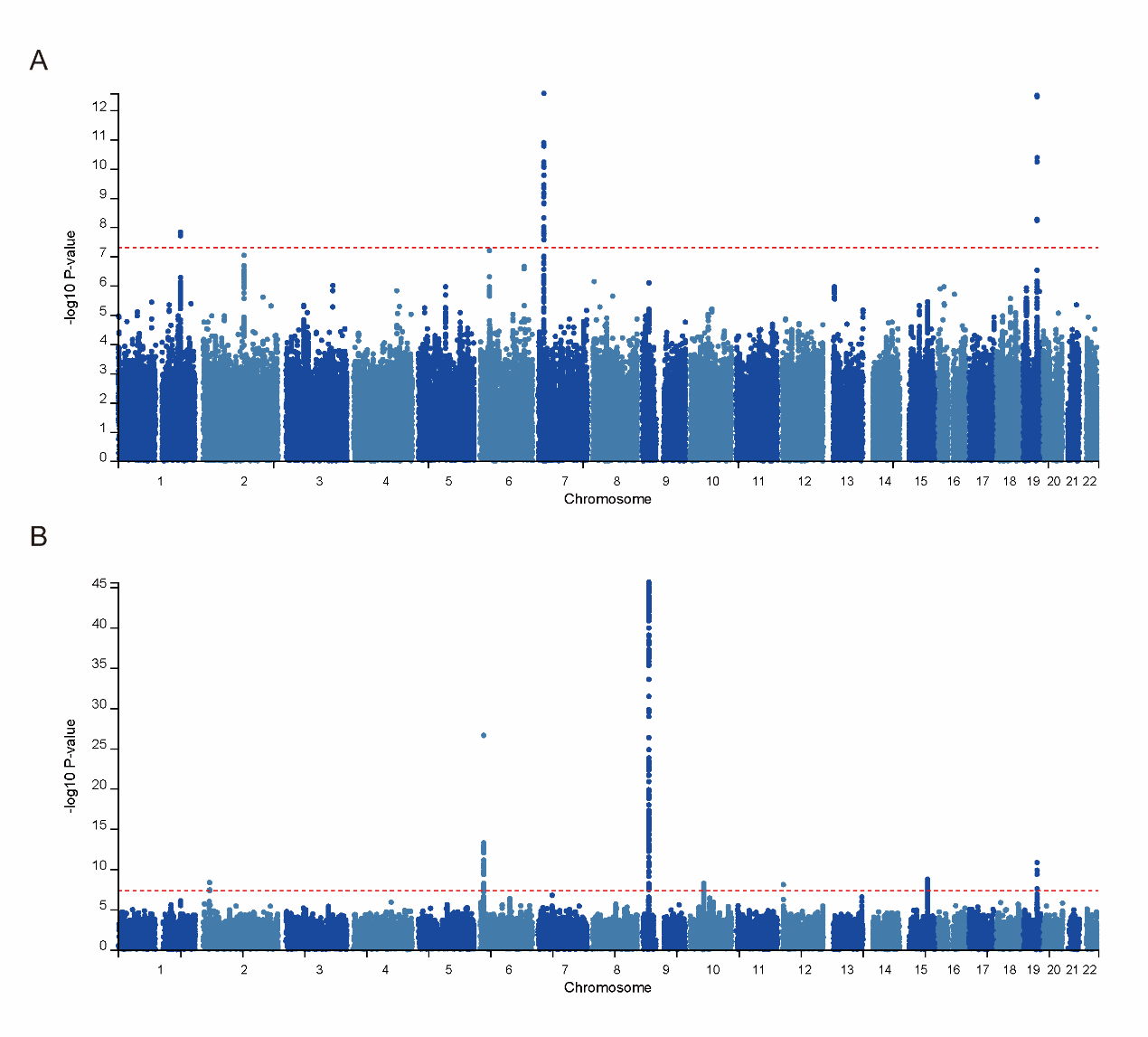

Supplement: Supplementary file 2 [file Data_Sheet_2.docx]
